# Remission of type 2 diabetes: position statement of the Italian society of diabetes (SID)

**DOI:** 10.1007/s00592-024-02317-x

**Published:** 2024-06-28

**Authors:** Danila Capoccia, Frida Leonetti, Andrea Natali, Domenico Tricò, Sebastio Perrini, Paolo Sbraccia, Valeria Guglielmi, On behalf of the Italian Society of Diabetes (SID)

**Affiliations:** 1 Department of Medical and Surgical Sciences and Biotechnologies, Sapienza University of Rome, Rome, Italy; 2 Department of Clinical and Experimental Medicine, University of Pisa, Pisa, Italy; 3 Department of Precision and Regenerative Medicine and Ionian Area, Section of Internal Medicine, Endocrinology, Andrology and Metabolic Diseases, University of Bari Aldo Moro, Bari, Italy; 4 Department of Systems Medicine, Unit of Internal Medicine – Obesity Center, Policlinico Tor Vergata, University of Rome Tor Vergata, Rome, Italy

**Keywords:** Type 2 diabetes (T2D), Obesity, Remission, Weight loss, Weight regain

## Abstract

The primary cause of the pandemic scale of type 2 diabetes (T2D) is the excessive and/or abnormal accumulation of adiposity resulting from a chronic positive energy balance. Any form of weight loss dramatically affects the natural history of T2D, favoring prevention, treatment, and even remission in the case of significant weight loss. However, weight regain, which is often accompanied by the recurrence or worsening of obesity complications such as T2D, is an inevitable biological phenomenon that is an integral part of the pathophysiology of obesity. This can occur not only after weight loss, but also during obesity treatment if it is not effective enough to counteract the physiological responses aimed at restoring adiposity to its pre-weight-loss equilibrium state. Over the past few years, many controlled and randomized studies have suggested a superior efficacy of bariatric surgery compared to conventional therapy in terms of weight loss, glycemic control, and rates of T2D remission. Recently, the therapeutic armamentarium in the field of diabetology has been enriched with new antihyperglycemic drugs with considerable efficacy in reducing body weight, which could play a pathogenetic role in the remission of T2D, not through the classical incretin effect, but by improving adipose tissue functions. All these concepts are discussed in this position statement, which aims to deepen the pathogenetic links between obesity and T2D, shift the paradigm from a “simple” interaction between insulin resistance and insulin deficiency, and evaluate the efficacy of different therapeutic interventions to improve T2D management and induce diabetes remission whenever still possible.

## Introduction

The concept of diabetes remission is driving a paradigm shift in our understanding of type 2 diabetes (T2D), no longer seen as an irreversible disease related to a progressive depletion of β-cells and various chronic diabetes-related complications, but rather as a disease that can be reversible through a personalized approach (nutritional, pharmacological, or surgical) primarily aimed at weight loss or reduction of ectopic fat predominantly contained in the liver, pancreas, and muscle. Obesity and T2D are indeed closely linked so that treating diabetes without addressing obesity becomes challenging. Specifically, studies on bariatric procedures which have differing efficacy on weight loss and T2D management [1], have paved the way for the concept of T2D remission, thus suggesting the hypothesis that T2D may be considered a reversible disease [2]. For this reason, this paper aims to analyze the pathogenetic links between obesity, prediabetes and T2D in order to assess the efficacy of different therapeutic interventions in inducing T2D remission whenever still possible.

## Obesity plays a central pathogenetic role in type 2 diabetes

**Fig. 1:**
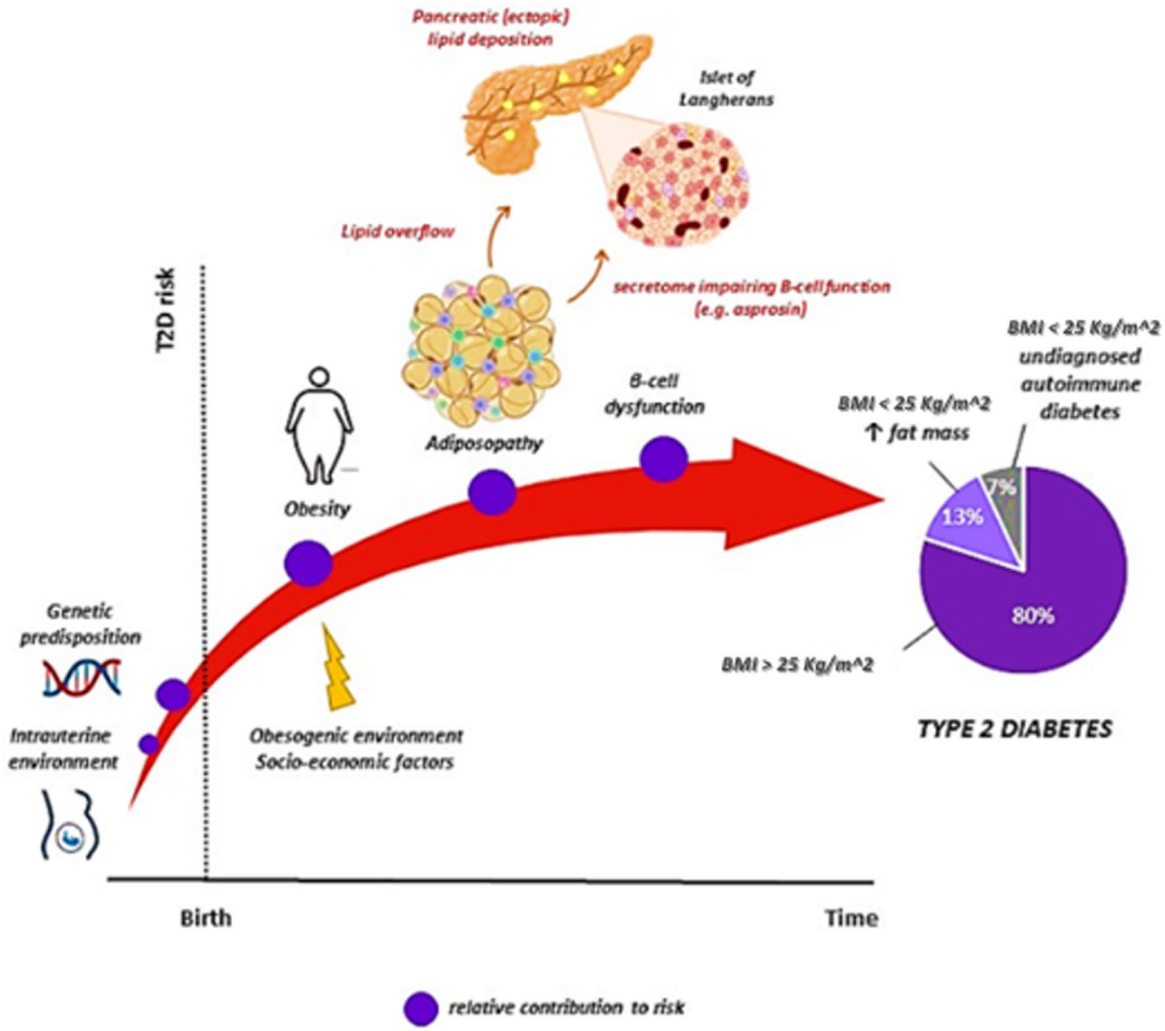
The relative contributions of adiposity-related factors to the pathogenesis of type 2 diabetes. Partially created with BioRender.com

The main cause of the pandemic scale of T2D is represented by excessive and/or abnormal accumulation of adiposity resulting from chronic positive energy balance (Fig. 1). About 80% of people affected by T2D have a BMI > 25 kg/m^2^; two-thirds of the remaining 20% have increased waist circumference and/or fat mass, while the remaining (true normal weight) show positivity to anti-insulin autoantibodies [3, 4]. Recently, a new classification of diabetes has been proposed and well received by the scientific community [4]; it involves five clusters based on age of onset, HbA1c, insulin resistance, indices of β-cell functionality, BMI, and the presence of autoantibodies in a cohort of over 20,000 patients with T2D one year after diagnosis. In a validation study using the cohort (over 7000 subjects) from the ORIGIN trial (Outcome Reduction with Initial Glargine Intervention), the mean BMI of all five groups was > 28 kg/m^2^ (with the minimum interquartile range > 25), including the “severe autoimmune diabetes” (SAID) group [5]. Furthermore, it has been recently demonstrated that adiposity in childhood is a risk factor not only for forms of diabetes characterized by obesity or insulin resistance but also for those characterized by insulin deficiency or autoimmunity [6, 7].

### Abdominal obesity is more closely associated with the risk of developing type 2 diabetes

Therefore, waist circumference or waist-to-hip ratio are better predictors of T2D than BMI. BMI has the major limitation of not providing information on body composition or fat distribution. Thus, for a given BMI value, the percentage of body fat and fat distribution can vary substantially. Abdominal obesity, defined by increased waist circumference values or waist-to-hip ratio, is associated with a greater risk of developing cardio-metabolic complications [8, 9]. This association can be attributed to the intrinsic metabolic properties of intra-abdominal fat (increased lipolytic and lipogenic activity, inflammation, and insulin resistance) and its anatomical localization, which justifies immediate and direct effects on the liver. More generally, intra-abdominal fat can also be understood as a marker of lipid accumulation in ectopic sites. Attention to abdominal fat distribution is particularly relevant for identifying those people who, despite being of normal weight, have increased waist circumference values and are therefore considered “living with metabolically obesity” or rather burdened with cardio-metabolic complications [3]. Adipocyte dysfunction is responsible for the development of metabolic complications of obesity [10, 11] (Fig. 1).

Just as in lipodystrophic syndromes, genetic or acquired conditions characterized by partial or generalized loss of fat, the progressive inability to accumulate excess calories leads to fat accumulation in ectopic sites (liver, pancreas, heart, etc.). In fact, the convergence of obesity and lipodystrophies on the same cardio-metabolic consequences has clearly allowed the understanding that adipose tissue also becomes dysfunctional in obesity and that this dysfunction plays a central role in the pathogenesis of the metabolic syndrome and T2D. At the basis of this dysfunction, now termed “adiposopathy”, there would be a complex sequence of morphological and functional alterations including reduction of adipogenic potential with prevalent expansion due to hypertrophy, recruitment of inflammatory cells, alteration of the secretion pattern of adipocytokines, inflammation, hypoxia, fibrosis, and mitochondrial dysfunction [12, 13].

Not all patients with excess fat mass develop T2D (although many have prediabetes) because the level at which adiposopathy is established is variable and based on various factors including central (visceral) vs peripheral (subcutaneous) distribution, sexual dimorphism, “adipocyte endowment” in childhood, and intrauterine and neonatal epigenetic imprinting as that observed in neonates with low and high birth weight who are both at increased risk of obesity and T2D [14, 15].

### Insulin resistance is a phenomenon secondary to chronically positive energy balance and progressive adipose tissue dysfunction

The race to discover the pathophysiological bases of insulin resistance begun in the second half of the last century, i.e., that primitive defect capable of determining compensatory hyperinsulinemia and causing T2D as β-cell response declines, has led to disappointing results [16]. Indeed, susceptibility loci for T2D identified through GWAS with few exceptions have been found to be associated either to a low risk of developing diabetes or mostly prone to affecting β-cell function rather than insulin sensitivity [17]. This was confirmed by the EPIC InterAct Case-Cohort Study which showed that obesity has a greater weight in defining the absolute risk of T2D than genetics [18]. Ectopic lipid accumulation promotes the development of insulin resistance through the formation of toxic lipid intermediates such as ceramides and diacylglycerol, which interfere directly and indirectly with insulin signaling and insulin-regulated metabolic pathways [19], as well as through altered secretion of insulin-sensitizing lipid signaling molecules (e.g., fatty acid esters) [20, 21]. This negative effect on insulin sensitivity seems to precede the development of local inflammation [22]. Other mechanisms implicated in the development of insulin resistance play a secondary, albeit important, role. Among these, inflammation, oxidative str1ss and endoplasmic reticulum stress, alteration of adipokine secretion pattern (particularly reduction in secretion of insulin-sensitizing adipokines such as adiponectin, omentin, and vaspin) and hormones, reduced catabolism of branched-chain amino acids, and release of exosomes carrying microRNAs capable of interfering with insulin signaling [20, 21].

### Insulin resistance represents a condition neither sufficient nor necessary for the development of type 2 diabetes

Insulin resistance is not sufficient for the development of T2D since the study of rare forms of primary insulin resistance has shown that T2D is not necessarily present in these cases [23]. Insulin resistance cannot be considered necessary in view of the results of the prodromal study for the DiRECT trial, which allowed a true paradigm shift and in particular to hypothesize that T2D is a reversible disease. Roy Taylor and his group have indeed demonstrated for the first time how in patients with obesity and T2D undergoing very low-calorie diet (VLCD), hepatic and pancreatic triglyceride content and blood glucose normalize after just one week, up to complete restoration of the first phase of insulin secretion after 8 weeks without any variation in insulin sensitivity [24]. This suggests that the progressive accumulation of lipids in the liver, which would trigger increased insulin secretion, and the accumulation of lipids in the pancreas, which would eventually lead to a reduction in β-cell function, play a central pathogenetic role in the development of T2D [25].

From this perspective, calorie restriction could reduce the risk of developing T2D, or promote its remission if already established, by increasing insulin secretion rather than alleviating insulin resistance [26]. Obesity itself, not insulin resistance, is responsible for hyperinsulinemia in people living with obesity.

Obesity is associated with increased plasma insulin concentrations both at baseline and postprandially. While it has been always believed that this increase was due to an adaptive response of β-cells aimed at compensating for altered insulin sensitivity, there is much evidence to support the hypothesis that β-cell hyperactivity, which can eventually lead to their failure, precedes the onset of insulin resistance. In fact, the increase in insulin secretion induced by obesity, along with a decrease in insulin clearance, is sufficient to explain the increase in plasma insulin concentrations needed to maintain normoglycemia [27].

Insulin resistance can be also interpreted as a (mal)adaptive response, at least initially aimed at countering excessive accumulation of energy metabolites in tissues [28, 29].

### Diabetes is a disease of the β-cell

Although peripheral insulin resistance is found in almost all patients with T2D, the development of hyperglycemia and disease progression are actually attributable only to a defect in insulin secretion. In this view, the seminal contribution of Clifton Bogardus and his group is valuable: in a now historic longitudinal study conducted on the Pima Indians, they demonstrated that the progression from a condition of normoglycemia to overt T2D is totally attributable to the reduction in insulin secretion [30].

## Prediabetes in people with overweight: proposal for a lifestyle intervention algorithm combined with new medications (not just metformin)

Prediabetes is the first sign of altered glucose metabolism, and its diagnosis can be placed on a continuum that places the patient at a high risk level for the future onset of T2D [31]. Prediabetes, indeed, presents an annual conversion rate to T2D of about 5–10% [32]. The diagnostic criteria for prediabetes are based on the relation of fasting glucose levels, glucose levels after OGTT and HbA1c with the risk of developing microvascular complications typical of T2D, especially diabetic retinopathy [23–35]. However, several clinical studies have shown that the diagnosis of prediabetes already defines a high cardiovascular risk independently of other risk factors [36, 37]. Therefore, the early recognition and treatment of this condition not only has the potential to prevent the onset of T2D but also to reduce cardiovascular mortality. Many studies have demonstrated the efficacy of lifestyle intervention [31, 38, 39] and some drugs approved for the treatment of T2D (metformin, thiazolidinediones, and acarbose) and obesity (orlistat) [32, 40] in preventing or delaying the progression of prediabetes to T2D, showing clearly how weight loss is the main mediator of this effect [38, 41]. In fact, in the Diabetes Prevention Program (DPP), metformin was less effective than intensive lifestyle intervention in reducing the risk of T2D (31% vs. 58%) in line with its lesser impact on body weight over the three years of the study [31, 42, 43]. Moreover, the evaluation of glycemic homeostasis at the time of its discontinuation clearly showed how its effect is mainly to mask rather than prevent the progression to T2D [44]. Finally, in the long term, as highlighted in the 20-year follow-up of the DPP (DPPOS, Diabetes Prevention Program Outcomes Study), neither metformin nor lifestyle intervention modified cardiovascular risk [38, 45] in line with previous studies [46–49]. From this, the clear need not only to intervene early in patients with prediabetes but also to identify new therapeutic strategies emerges. In this context, it is believed that the use of GLP-1 receptor agonists (GLP1-RA) associated with lifestyle intervention already in the early stage of the disease may help prevent the progression of β-cell dysfunction, thanks to the action of these drugs on body weight and probably their direct effect on β-cells [50]. The SCALE Obesity and Prediabetes study has indeed demonstrated the efficacy of Liraglutide 3 mg in addition to lifestyle intervention not only in the treatment of obesity but also in terms of T2D prevention in people with prediabetes and living with overweight/obesity [51]. The results show a 66% reduction in the risk of T2D at three years compared to placebo treatment [52]. In the STEP-1 and STEP-5 studies, semaglutide 2.4 mg led to the restoration of normoglycemia in 84% and 80% of subjects with prediabetes (compared to 48% and 37% in the control group), respectively after 68 and 104 weeks of treatment, and reduced the risk of progression to T2D [53]. Similarly, in the recent OASIS-1 study, 76% of participants with prediabetes achieved normalization of blood glucose after 68 weeks of treatment with oral semaglutide 50 mg once a day, compared to 10% of subjects with prediabetes in the placebo group [54]. Moreover, 5 randomized clinical trials (RCTs) with cardiovascular outcomes have shown the cardiovascular and renal benefits of some drugs in the GLP1-RA class (liraglutide, semaglutide, albiglutide, dulaglutide, efpeglenatide) in subjects with T2D at high cardiovascular risk [55], which could also extend to subjects with prediabetes. In fact, the cardiovascular protection of these drugs seems to depend on direct and indirect mechanisms at least partially independent of their hypoglycemic and anorectic action, of variable entity and in some cases modest [56]. Further developments in pharmacotherapy for the simultaneous treatment of prediabetes associated with overweight may also derive from the promising results of early studies on the use of new incretin receptor coagonists with combined action on GLP-1, GIP, and/or glucagon receptors, which have shown greater efficacy in glycemic control and weight loss compared to selective agonists. In the SURMOUNT-1 study, in fact, over 95% of participants with prediabetes were able to achieve normalization of blood glucose after 72 weeks of treatment with tirzepatide, the first GIP/GLP-1 receptor co-agonist representing this new class of drugs, compared to 62% of the control group [57]. Similarly encouraging are the results of a phase 2 study on the triagonist receptor agonist retatrutide showing normalization of blood glucose in 72% of subjects with prediabetes after 48 weeks of treatment compared to 22% of the control group [58]. The full recognition of prediabetes as a disease has also been advocated in a recent Italian consensus of experts proposing to include prediabetes among the complications of obesity that allow, when present, the prescription of drugs for the treatment of obesity even in patients living with overweight (BMI ≥ 27 kg/m^2^) [59]. A more in-depth study of the mechanisms of prediabetes remission, its duration following discontinuation of pharmacological therapy, and the short- and long-term cardiovascular benefits deriving from normalization of glycemic control will be necessary to allow adequate evaluation of the feasibility and cost–benefit ratio of these interventions.

## Nutritional strategies: lessons from look AHEAD, DIRECT, ReTUNE

Weight loss, however achieved, dramatically impacts the natural history of T2D, favoring prevention, treatment, and even remission. Therefore, international guidelines recommend that all people with T2D and living with overweight or obesity are supported with evidence-based strategies, primarily dietary, aimed at achieving and maintaining significant weight loss [60]. To date, available experimental evidence supports the paradigm that every weight loss achieved through dieting may be followed by diabetes remission in a proportionally large number of patients, while it is less clear whether qualitative aspects of the diet are equally influential.

Recent systematic reviews and meta-analyses identified the use of very low-calorie diets, providing 500–800 kcal per day and often employing artificial liquid formulas to partially or totally replace meals, as the most effective nutritional strategy in promoting weight loss and potential disease remission in people with diabetes [61]. The first experimental evidence in this regard comes from a secondary analysis of the Look AHEAD study, which evaluated the effectiveness of a hypocaloric diet with the use of liquid meal replacements within the context of an intensive lifestyle intervention in terms of diabetes remission [62]. The study results showed a weight loss of 8.6% compared to baseline body weight in the intervention group, corresponding to a one-year diabetes remission rate of about 12%. The differences between groups gradually diminished during the 4-year follow-up, as expected, but never disappeared completely. Twelve years later, participants in Look AHEAD who had achieved diabetes remission, albeit temporary, showed a 40% reduction in cardiovascular disease risk and a 33% reduction in chronic kidney disease risk, which increased to 49% and 55% in case of sustained remission for at least 4 years [63].

Based on these promising results, the DiRECT study was designed and conducted, the first randomized, controlled, multicenter trial with the primary endpoint being diabetes remission achieved through dietary intervention in people living with overweight (BMI 27–45 kg/m^2^) with a short duration of disease (< 6 years) [64]. The DiRECT study intervention included an intensive weight loss induction phase lasting three months with total diet replacement using artificial liquid meals and discontinuation of all hypoglycemic medications, a gradual reintroduction phase of solid foods lasting two months, and then a maintenance phase [65]. At one year, one quarter of subjects in the intervention group achieved the target weight loss of 15 kg (mean weight loss approximately 10 kg), and almost half achieved diabetes remission [64]. The diabetes remission rate remained significantly high even after two years of intervention, albeit decreasing to 35.6% in parallel with weight regain [66]. The efficacy of a dietary intervention similar to that proposed in DiRECT was subsequently confirmed by the DIADEM-I study, which demonstrated a remission rate even higher, at 61%, in a younger population with a shorter disease history [67], and by the DiRECT-Aus study, which replicated the results of DiRECT in the Australian population [68].

In these studies, the actual achieved weight loss was identified as the greatest predictor of diabetes remission. In fact, an exploratory analysis of the DiRECT study showed how the remission rate increases directly proportional to weight loss, approximately 5–6% for every kg lost, reaching 86% at one year and 70% at two years in those who achieved the target weight loss of at least 15 kg [66]. Conversely, in DiRECT, no significant effect of baseline BMI on the chances of diabetes remission was observed. This unexpected finding could be justified by the poor ability of BMI alone to recognize some obesity phenotypes associated with altered distribution of body fat and presence of metabolically unfavorable ectopic fat, in the absence of significant weight gain [69–71]. To demonstrate this hypothesis, the ReTUNE study was conducted, involving 20 patients with diabetes and intrahepatic and/or intrapancreatic fat accumulation, measured by abdominal resonance, despite a BMI indicative of normal weight or mild overweight (< 27 kg/m^2^) [62]. Patients underwent 1–3 cycles of weight loss of 5% compared to initial body weight, each comprising 2–4 weeks of hypocaloric diet of approximately 800 kcal/day with liquid meal replacements and vegetables, and 4–6 weeks of maintenance. At 12 months of follow-up, in the face of a mean weight loss of 10.7% (7.7 kg), diabetes remission was observed in 14 subjects (70%) and a reduction in total fat mass of 14%, accompanied by a more marked reduction in intrahepatic (− 63%) and intrapancreatic fat (18%).

Less solid data are available in the literature supporting the effectiveness of qualitative dietary interventions on weight loss and disease remission in people with T2D [61]. Imbalanced popular diets low in carbohydrates or high in protein, as well as ketogenic diets, do not seem to be superior to more conventional diets, and for this reason, European recommendations from the Diabetes Nutrition Study Group do not support their use for weight loss purposes [60]. More balanced diets, including the vegetarian diet and the Mediterranean diet, could instead lead to some benefit in people living with T2D in terms of weight loss and, perhaps, disease remission [61]. In fact, despite the lack of dedicated studies, a secondary analysis of an Italian clinical trial showed how the Mediterranean diet is capable of inducing remission at one year greater than 10% in subjects with newly diagnosed T2D, with a weight loss of only 2 kg greater, compared to a conventional low-fat diet [72]. This data suggests that the peculiar qualities of the Mediterranean diet (or some specific characteristics of the Italian population) may confer an additional benefit in terms of weight loss-induced disease remission and would confirm the absence of a threshold effect, whereby T2D remission would be achievable even in the face of relatively modest weight loss. Despite the overwhelming superiority of scientific evidence in support of the effectiveness of very low-calorie diets with total meal replacement in inducing diabetes remission, especially in selected populations, a less intensive approach, such as that offered by the Mediterranean diet, could offer a valid alternative for patients motivated to pursue disease remission but unwilling to completely change dietary habits, perhaps in the context of a non-invasive multidimensional intervention against obesity that includes exercise, medication, and cognitive behavioral therapy.

## The present and future role of pharmacological treatments for type 2 diabetes with significant effects on weight: early/innovative place in therapy

In recent years, the therapeutic armamentarium in the field of diabetology has been enriched with various classes of drugs, allowing for a broader choice with the possibility of therapy customization. In addition to identifying a specific HbA1c target for each patient, it is recommended to choose the most appropriate drug based on their comorbidities, taking into account the evidence of cardiovascular and renal benefits of some drug classes regardless of their effect on glycemia [73].

However, weight loss of 5–15% should be a primary goal in patients with T2D because it not only improves glycemic control and quality of life but also prevents or treats further cardio-metabolic complications of obesity [74]. In particular, a 5–10% weight loss confers significant metabolic benefits, while a weight loss of at least 10–15% can lead to diabetes remission [74, 75]. GLP1RA and GLP1/GIP RA are currently the only approved antihyperglycemic drugs that allow patients with T2D to achieve weight loss percentages ≥ 10% (in 28.7% and 45.6% of subjects with semaglutide 1 mg and 2.4 mg [76] and in 60.5% and 64.8% of subjects with tirzepatide 10 mg and 15 mg, respectively [77] and ≥ 15% (in 13.7% and 25.8% with semaglutide 1 mg and 2.4 mg [76] and in 39.7% and 48% with tirzepatide 10 mg and 15 mg, respectively [68]. Although the effect on glycemic control of these drugs is partly independent of their effect on body weight [53], the latter remains a fundamental goal for treating obesity and its complications and promoting T2D remission where weight loss > 15% is achieved. Furthermore, the cardiovascular protective effects of liraglutide at a maximum dosage of 1.8 mg demonstrated in the LEADER study over a median follow-up of approximately 4 years are attributed not only to the improvement of glycemic and metabolic compensation, lipid profile, and blood pressure but also to the reduction of visceral and ectopic fat induced by liraglutide, as demonstrated in a study conducted on people living with overweight or obesity treated with liraglutide 3 mg [78].

In this perspective, it is highlighted the need not only for the use of GLP1-RAs or GLP1/GIP-RA with a more pronounced effect on body weight as first-line drugs (semaglutide, tirzepatide) also simultaneously wo metformin similarly to what is already in place in the treatment of hypertension [79], but also for careful stratification of patients with T2D based on BMI or waist circumference in order to select those who would derive greater benefit from the use of these drugs at the approved dosage for obesity treatment (semaglutide 2.4 mg) under reimbursement schemes. In addition to this, the recently published results of the SELECT study endorse semaglutide as a new weapon for cardiovascular prevention. This study has shown that subcutaneous once-weekly semaglutide 2.4 mg, was associated with a significant 20% reduction in major adverse cardiovascular events (MACE) compared with placebo in people living with overweight/obesity and established cardiovascular disease (CVD) but without diabetes [80].

A frontier of research is the development of triple receptor agonists (GLP1/GIP/glucagon-RAs) that combine the efficacy of GLP1/GIP-RAs on T2D and the efficacy of GLP-1/glucagon-RAs on obesity. Among these, retatrutide emerges on the horizon which from preclinical and phase 2 studies and appears to surpass the efficacy of GLP1/GIP receptor co-agonism [58, 81].

In this promising context, the likely availability in the near future of drugs for T2D at dosages equal to those used in obesity and with average weight reduction of ≥ 20% compared to baseline, such as tirzepatide 15 mg [77], oral semaglutide 50 mg [54], CagriSema 2.4/2.4 mg [82], will compellingly raise the issue of first-line treatment of patients with T2D, which should clearly be addressed to the fundamental pathogenetic moment of T2D, namely the increase in adiposity.

## Remission of diabetes after bariatric surgery as a proof-of-concept result

An increasing number of controlled and randomized studies suggest a superior efficacy of bariatric surgery compared to conventional therapy in terms of weight loss, glycemic control, and rates of T2D remission [83]. The main surgical procedures are divided into restrictive ones, such as adjustable gastric banding (AGB) that uses an adjustable silicone ring to reduce gastric capacity, vertical sleeve gastrectomy (VSG), which involves removing most of the stomach, reducing it to a vertical gastric sleeve, and mixed restrictive-malabsorptive procedures such as Roux-en-Y gastric bypass (RYGB), in which a small gastric pouch is created and anastomosed to a jejunal loop, and biliopancreatic diversion (BPD) which involves resecting the distal stomach anastomosed to the final segment of the small intestine [84]. Undoubtedly, the more anatomically complex the bariatric surgery and the greater the malabsorptive component, the higher the rate of diabetes remission. Additionally, preoperative parameters such as younger patient age, higher preoperative BMI, and especially the duration of diabetes, particularly less than 8–10 years, are key factors predicting a higher likelihood of diabetes remission after metabolic bariatric surgery [85].

### Criteria for diabetes remission after bariatric surgery

In 2021, the American Diabetes Association (ADA) modified the definition of diabetes remission by evaluating only the HbA1c value, which must be < 6.5% (7.8 mmol/l) for at least 3 months after surgery and hypoglycemic therapies, excluding fasting blood glucose measurement [86]. In patients undergoing bariatric surgery, the improvement in metabolic status has led to the use of the term “metabolic surgery—MBS,” identifying it not only as a simple surgical intervention for weight loss but also as a metabolic intervention capable of treating obesity-associated comorbidities, or even inducing their remission, such as T2D [87]. In this case, there is almost an 80% remission of diabetes cases in the first 2 years with maintenance over time in at least four out of five patients. Where diabetes persists after surgery, therapeutic management allows glycemic targets to be achieved with simplification of pharmacological therapy [88]. The 2020 American Diabetes Association (ADA) guidelines for managing diabetes in patients with obesity state that MBS should be recommended in patients with class III obesity (BMI ≥ 40 kg/m^2^) regardless of their glycemic control level, in patients with class II obesity (BMI ≥ 35 kg/m^2^) who show inadequately controlled diabetes despite lifestyle and optimal medical treatment, and even recommended in adults with T2D and BMI between 30 and 34.9 kg/m^2^ if weight loss or improvement in comorbidities is not achieved with pharmacological therapy [85]. Specifically comparing the two most commonly used forms of bariatric surgery, RYGB and VSG, the STAMPEDE study conducted on patients with obesity and T2D showed similar efficacy in glycemic control between the two procedures, albeit with less pharmacological therapy in the RYGB group [89]. Although the role of MBS in T2D remission is not yet fully clear, in recent years the existence of multifactorial mechanisms has been proposed [90]. In fact, if conventional theories recognized weight loss as the main mechanism for resolving T2D after MBS, the early improvement in glycemia immediately after surgery and even before weight loss suggested a potentially weight-independent glycemic control mechanism, especially in procedures requiring GI tract modeling like RYGB or VSG. In summary, the hypoglycemic effect induced by MBS can be depicted as an early effect, given by the reduction in gastric volume and therefore nutrient absorption, improvement in hepatic insulin response, improvement in gastrointestinal hormones secretion, and a late effect dependent on weight loss contributing to the maintenance of T2D remission over time [88]. The reduction in total, visceral, and pancreatic adipose tissue improves long-term insulin sensitivity, particularly in muscle and adipose tissue, while regarding the improvement in hepatic insulin sensitivity, the liver seems to be more susceptible to weight-independent effects [89]. Since the main causal factor of T2D in obesity is impaired β-cell function, this is precisely alleviated after MBS, and its alteration has been increasingly considered central to T2D remission [88].

### “Twin-cycle hypothesis”

As described earlier, obesity itself, not insulin resistance, is responsible for hyperinsulinemia in people with obesity. In fact, it was the theory of twin cycles or “Twin-cycle hypothesis” that hypothesized that T2D is mainly caused by the vicious cycle of excessive, but reversible, accumulation of fat in the liver and pancreas [82]. Therefore, reducing lipid deposition in these tissues and organs is important for diabetes remission [86]. In obesity, chronic inflammation of the islets, and glucotoxicity resulting from high basal insulin levels, would cause progressive β-cell dysfunction, inducing apoptosis and cellular dedifferentiation, identified as the central trigger for the development of T2D [88]. The removal of excess fat from these organs, secondary to weight loss, is associated with recovery by β-cells of acute insulin secretion after oral glucose load tests [91], suggesting that the process of redifferentiation may underlie the return to β-cell function [92]. Improved insulin sensitivity in the liver contributes to restoring normal hepatic glucose production during fasting, reducing the toxic effect of hyperglycemia on β-cells. Moreover, rapid recovery of β-cell function and mass and the first phase of insulin secretion after RYGB and VSG seem to contribute to rapid improvement in glycemic homeostasis aided by the implementation of gastrointestinal hormone secretions obtained with various interventions [87].

### “Foregut hypothesis”

Numerous studies have observed that increased secretion of some gastrointestinal hormones enhances β-cell function after bariatric surgeries [88], hypothesizing that T2D remission may result from a faster passage of food at the distal small intestine where L cells are concentrated (“foregut hypothesis”). In fact, in the posterior intestine, these cells are responsible for the release of intestinal peptides implicated in weight loss and stimulation of insulin secretion, such as GLP-1 and peptide YY (PYY) [87]. The increase in postprandial GLP-1 levels, up to 10 times within a few days of surgery, is thought to contribute to the improvement of glucose homeostasis already in the early postoperative period [89]. This effect is amplified by increased postprandial glucose levels due to increased food transit speed [93]. GLP-1 also seems to stimulate insulin biosynthesis by implementing proinsulin gene transcription. PYY levels also increase after MBS, such as RYGB and VSG, contributing to restoring normal insulin response to glucose in the postprandial phase [87].

### Role of bile acids and intestinal microbiota

Due to the alterations induced on the enterohepatic circulation, plasma levels of bile acids are also increased after MBS, and this increase allows bile acids to act as signaling molecules in multiple target organs [94]. Once in the intestine, these seem to activate the G protein-coupled receptor 5 (TGR5) expressed on L cells, stimulating the release of GLP-1 and consequently, indirectly improving glucose metabolism and food intake. In addition, within the intestine, luminal bile acids also seem to interact with the farnesoid X receptor (FXR), increasing the expression of fibroblast growth factor 19 (FGF19) which in turn acts on the liver by decreasing gluconeogenesis and lipogenesis [95]. Finally, since alterations in the intestinal microbiome are implicated in the development of metabolic complications, such as obesity, food intake, and hyperglycemia, it has been observed that modification of the intestinal microbiota following MBS contributes to weight loss, changes in bile acid metabolism, and also long-term T2D control rates [96]. For example, the Firmicutes genus (dominant in people living with obesity) significantly decreased in people after MBS, associating with significant weight loss, improved insulin sensitivity, and widespread systemic inflammation, while the Akkermansia genus (reduced in mice with obesity and T2D) increased after MBS, showing reduced fat mass, increased thermogenesis, and induction of systemic GLP-1 secretion in mice [87]. Further studies are needed to clarify to what extent the intestinal microbiome improves glucose metabolism after an MBS intervention. Overall, while the combination of GLP-1 release with calorie restriction improves β-cell function in the early postoperative period and weight loss plays a primary role in improving peripheral insulin sensitivity in the late postoperative period (between 3 and 6 months after surgery), long-term improvement in insulin sensitivity and body weight has been primarily attributed to increased postprandial levels of anorexigenic gastrintestinale hormones (GLP-1 and PYY). These hormones indeed promote greater satiety and allow for maintaining reduced caloric and food intake. However, it is important to consider that preoperative pancreatic reserve, and therefore the duration of diabetes, are key factors in predicting postoperative glycemic control improvement and therefore T2D remission [97]. To this end, the use of scores such as ABCD, IMS, and DiaRem guide the prediction of response in terms of T2D remission, confirming that parameters such as patient age, preoperative BMI, HbA1c, insulin therapy use, and especially diabetes duration and C-peptide levels play a key role in T2D remission after bariatric surgery [98].

Finally, it is important to emphasize that even in cases where complete remission of diabetes does not occur, significant improvement in glycemic control is still achieved after the intervention and therefore a lower risk of onset and/or worsening of chronic complications of the disease is observed.

## Diabetes management in bariatric patients

The management of diabetes in bariatric patients has peculiar aspects both in the pre and postoperative period. Current guidelines lack specific indications regarding adjustments of hypoglycemic medications before bariatric surgery, leading various authors to provide recommendations solely based on clinical experience. To date, there is not much evidence indicating specific glycemic targets to achieve in the preoperative phase, but much of the literature agrees that a reduced value of preoperative glycosylated hemoglobin may predict greater weight loss and a lower incidence of peri- and postoperative complications. In this phase, modifications of hypoglycemic therapy are therefore necessary to optimize glycemic control and promote an initial weight loss (5–10%), which most bariatric programs strongly suggest to improve metabolic parameters and liver volume, facilitating laparoscopic interventions [99].

In post-bariatric patients, modifications of antidiabetic therapy are necessary immediately after the intervention due to the drastic reduction in caloric intake and rapid changes in insulin sensitivity. The use of one class of drugs over another is still poorly supported by guidelines. What is widely shared among experts suggests that, given the chronic nature of diabetes, careful monitoring is necessary, including closer follow-up visits in the first and second years post-intervention, and subsequently annual measurement of glycated hemoglobin even in the presence of complete remission. Adequate education on self-monitoring in patients who remain living with diabetes should also be recommended, especially concerning the type of surgical intervention and the possibility of postprandial hypoglycemia, especially in gastric bypass.

Bariatric patients without diabetes have lower mortality after surgery, while patients with T2D who achieve remission after surgery have a reduced risk of mortality compared to patients who do not go into remission. Given the significant evidence obtained from new pharmacological classes in reducing cardiovascular mortality, it is clear that their use in the post-intervention period deserves consideration. The optimal timing to start antidiabetic medications after bariatric surgery, as well as the choice of pharmacological class, remains a matter of debate.

### SGLT2 inhibitors

It is known that SGLT2 inhibitors (SGLT2i) are associated with an increased risk of diabetic ketoacidosis [100], with the particularity that this adverse event generally occurs in the presence of modest hyperglycemia compared to the classic ketoacidosis of patients with type 1 diabetes. The explanation for this so-called euglycemic ketoacidosis is to be found in the fact that SGLT2i promote glucosuria. Surgeries in general, and abdominal ones in particular, can increase the risk of ketoacidosis due to the release of hyperglycemic hormones (glucagon, cortisol), significant caloric restriction, or inadequate management of insulin therapy. Bariatric/metabolic surgery accentuates all these risk factors, and low caloric and glycemic intake can further increase the onset and progression of ketoacidosis. In a systematic literature review [101], which analyzed a total of 36 studies reporting cases of euglycemic ketoacidosis associated with SGLT2i therapy after surgery, 13 cases out of 42 concerned bariatric surgery. The fact that bariatric surgery is one of the most important risk factors for perioperative SGLT2i-induced ketoacidosis has been recently confirmed by another systematic review of 99 case reports [102], where the Authors advocate for a desirable discontinuation of SGLT2i at least three days before surgery. SGLT2i are known to promote weight loss, and all the most recent randomized case–control trials have demonstrated a lower incidence of new cases of diabetes in IGT (Impaired Glucose Tolerance) subjects treated with SGLT2i compared to placebo; whether this positive result can be extrapolated and applied to patients with T2D who experience remission (transient) after bariatric/metabolic surgery remains completely unproven.

Effectiveness of SGLT2i on postprandial hypoglycemia is a complex and multifactorial complication of bariatric surgery, which can be asymptomatic or associated with severe symptoms and may require, in addition to dietary treatment, also the use of medications. SGLT2i have provided promising results in this field. Three different studies have suggested that dapagliflozin, canagliflozin, and empagliflozin may be useful in limiting the risk of post-bariatric hypoglycemia [103]. Dapagliflozin 10 mg/day produced symptomatic improvement of the hypoglycemic syndrome associated with both hyperglycemia and hypoglycemia documented during glucose self-monitoring. A pilot study conducted on 21 subjects [104] with postprandial hypoglycemia showed that canagliflozin (300 mg/day) significantly reduced blood glucose and insulin levels 60 min after a meal following the intake of 100 g of glucose during an oral glucose tolerance test in patients with previous RYGB. A third study in 14 patients (9 without T2D and 5 with T2D) showed a significant increase in blood glucose, with no significant changes in plasma insulin levels with the use of empagliflozin 25 mg, an effect attributed to an increase in hepatic glucose production [105]. Another study demonstrated that acute inhibition of SGLT1/SGLT2 with canagliflozin (high dose of 600 mg) in 10 patients treated with RYGB attenuated the early increase in GLP-1, GIP, and plasma insulin after glucose ingestion (50 g), with a later increase in glucagon concentrations, producing combined effects that could lead to a lower risk of postprandial hypoglycemia [106]. Finally, an interesting mechanistic study concluded that the increase in interleukin-1 (IL-1) induced by hyperglycemia may play a role in the onset of postprandial hypoglycemia after gastric bypass. Empagliflozin could limit this complication, by reducing secretion of insulin and of a selective IL-1 antagonist named “anakinra” [107].

### Glucagon-like peptide-1 receptor agonists

Given the proven efficacy of GLP1-RA in promoting weight loss, this class of drugs would find a good place in managing bariatric patients with weight regain and also in patients with pre-existing diabetes or recurrence after surgery. Up to 50% of patients taking GLP1-RAs experience side effects such as nausea, vomiting, and diarrhea, which tend to self-limit and resolve over time [108]. Since these symptoms overlap with the initial disturbances of the immediate postoperative period, it would be prudent not to introduce this class of drugs into therapy until operated patients reach the solid phase of the post-intervention diet. Regarding the efficacy of these molecules on weight regain after bariatric surgery, the data available today are limited to observational studies or case reports with liraglutide. The GRAVITAS study, a randomized controlled trial, on patients undergoing bariatric surgery with persistent or recurrent T2D, showed that patients treated with liraglutide at a dosage of 1.8 mg/day achieved a significant reduction in HbA1c and body weight [109]. Experiences with the use of semaglutide (injectable or oral) in the postoperative period are very limited; a retrospective analysis of 44 patients with insufficient weight loss or weight regain showed that patients treated with semaglutide achieved a weight loss of 10.3% after 6 months of treatment at a dosage of 0.5 mg, significantly lower than the 1–2 mg weekly doses used in trials with semaglutide as monotherapy. Another recent retrospective and monocentric study [110] evaluated the effectiveness of liraglutide and semaglutide in reducing body weight in 50 patients with weight regain after bariatric surgery. The average weight loss with the use of GLP1-RAs for 6 months in patients with previous bariatric surgery was 8.8% (higher with semaglutide than with liraglutide), resulting in a reduction of about two-thirds of the regained weight. Side effects were mainly related to gastrointestinal disturbances and were mostly mild and transient. The extent of weight loss observed with GLP1-RAs in this population of real-life patients is consistent with the results from the SCALE, SUSTAIN, and STEP clinical trials, which are based on patients without bariatric surgery.

The rationale for using this class of drugs after bariatric surgery lies in the pathophysiological changes induced by surgical procedures. As known, the rapid passage of nutrients at the level of intestinal L cells after surgery leads to increased secretion of intestinal hormones, especially postprandial GLP-1 and peptide YY. Both hormones are known to reduce food intake by stimulating satiety and decreasing appetite, which likely plays a central role in the weight loss achieved with bariatric surgery [111]. Some studies indicate that the hormonal changes observed, such as endogenous GLP1 secretion, after bariatric surgery may diminish over time [112], supporting the thesis that exogenous stimulation of GLP-1 receptors with GLP1-RA may have a greater effect on weight loss.

### DPP-IV inhibitors

DPP-IV inhibitors are generally very well tolerated, do not cause hypoglycemia, and are weight-neutral. An RCT, lasting 4 weeks, evaluated the efficacy of sitagliptin in patients with T2D after RYGB, reporting a significant reduction in blood glucose in the absence of side effects [113]. Due to their good safety profile, essentially devoid of side effects, DPP4 inhibitors are suitable pharmacological agents for post-bariatric patients with mild hyperglycemia; however, they would not be appropriate as monotherapy for significant hyperglycemia or to induce weight loss due to only modest improvement in A1c (0.7%) and no impact on weight.

### Metformin

Despite the widespread use of metformin, few studies have evaluated its efficacy and safety in the perioperative period of bariatric surgery. Metformin does not cause hypoglycemia and can be continued throughout the preoperative period of caloric restriction leading to preoperative weight loss. All surgical guidelines recommend discontinuing metformin 24–48 h before surgery due to the well-known risk of lactic acidosis [114]; these same recommendations are clearly extended to bariatric surgery. Metformin administration in the perioperative period requires adequate dose adjustments since it has been shown that metformin absorption increases by 50% after bariatric surgery [115]. Due to the known gastrointestinal side effects, it would be appropriate not to introduce metformin in the immediate postoperative period where gastrointestinal disturbances could overlap and confuse.

### Insulin

Patients on insulin therapy who begin a preoperative pathway with initial reduction of caloric intake may already undergo a reduction in insulin requirements and frequent dose adjustments to eliminate the risk of hypoglycemia. Patients should be educated on self-monitoring to achieve desirable fasting glycemic targets between 80 and 110 mg/dl through adequate titration of basal insulin (whose quantity generally needs to be reduced by 20%). The reduction in caloric and glycemic intake and weight loss lead to rapid improvement in insulin sensitivity. For patients on fixed doses of prandial insulin, a 20% reduction in insulin is recommended with subsequent adjustments based on postprandial blood glucose levels until eventual discontinuation if postprandial blood glucose remains below 140 mg/dl [116].

### Sulfonylureas

It remains the shared position of all guidelines not to start and to discontinue all sulfonylureas (SUs) in patients with T2D. The main concerns associated with the use of SUs are mainly related to hypoglycemia and weight gain, conditions that contraindicate their use in patients intending to undergo or who have undergone bariatric surgery. No studies have been conducted using SUs in patients post-bariatric surgery, and especially thanks to the availability of many other T2D drugs that do not induce weight gain or increase the risk of hypoglycemia, SUs are never preferred for the management of T2D in the perioperative or postoperative period.

Managing patients with pre-existing diabetes or with a new diagnosis of diabetes following bariatric surgery requires particular management skills due to potential adverse effects and the well-known strengths of available pharmacotherapy. Therapies should be aimed at reducing hyperglycemia with the fewest adverse effects, including weight gain. Finally, clinicians should pay particular attention during the early postoperative period when the risks of dehydration, ketoacidosis, and hypoglycemia are higher.

## Pathophysiology of weight regain

**Fig. 2:**
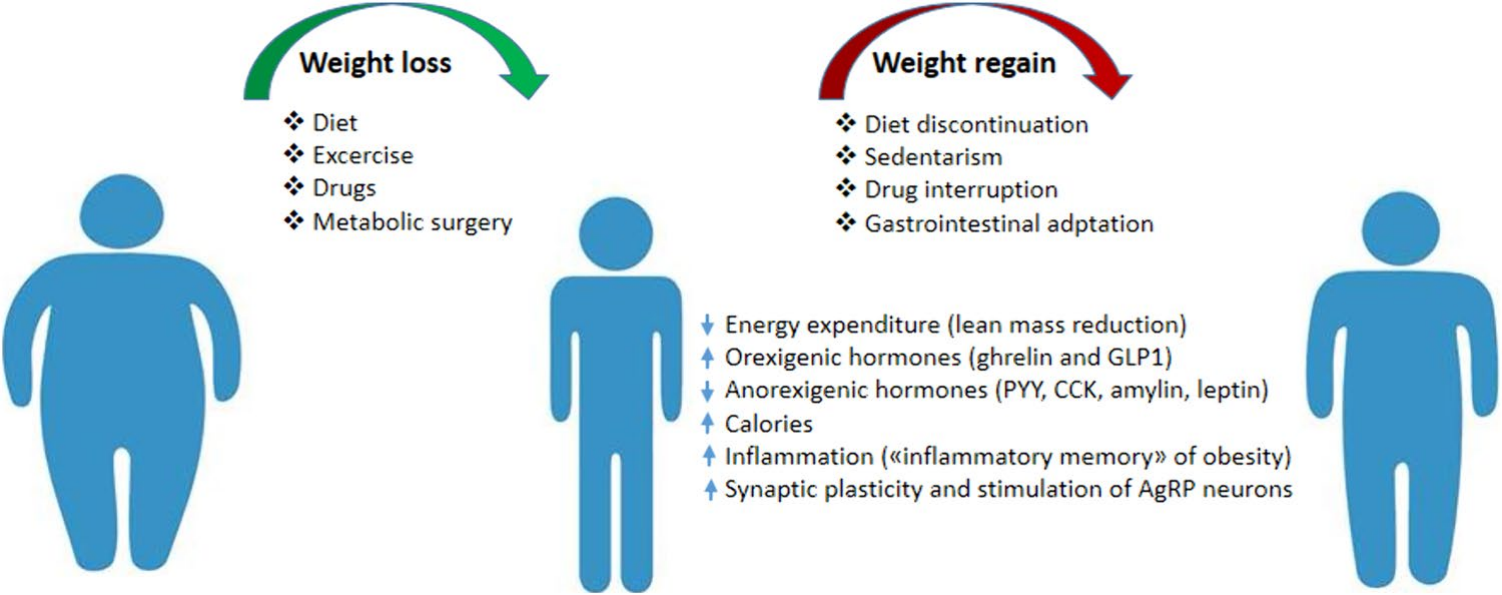
Mechanisms underlying the weight regain

Weight regain after weight loss is an unavoidable biological phenomenon in obesity as it is an integral part of its pathophysiology and expression of its relapse (Fig. 2). This can occur not only upon cessation but also during obesity treatment [117] when it is not sufficiently powerful and effective in counteracting the set of physiological responses aimed at restoring adiposity to the pre-weight loss equilibrium state (referred to as “set point”) [118, 119]. Weight regain is often accompanied by the reappearance or worsening of obesity complications [120].

Obesity relapse has often been attributed to poor patient adherence to treatments. Conversely, it is now widely recognized that body weight and adiposity are finely regulated by numerous physiological mechanisms that go well beyond people will.

Although the mechanisms underlying the tendency to regain lost weight are still largely unknown, it is believed that weight regain is caused by metabolic adaptations to weight loss, represented by an increase in orexigenic hormones such as ghrelin and GLP-1, and a concomitant reduction in anorexigenic hormones such as PYY, cholecystokinin (CCK), amylin, and leptin [121] even compared to normal-weight subjects. These compensatory variations would lead to increased appetite, craving, orosensory sensitivity, and hedonic value of food, resulting in a consequent increase in caloric intake [122, 123], as also suggested by a lesser attenuation of brain activity after food intake evaluated by functional MRI in subjects with weight regain compared to those who maintained lost weight [124]. Weight loss also appears to affect the synaptic plasticity of glutamatergic neuronal systems responsible for stimulating orexigenic AgRP neurons, thus enhancing their activity [125].

Another metabolic adaptation capable of promoting weight regain is represented by the reduction in energy expenditure [126, 127]. Basal metabolism after weight loss is indeed lower than expected based on changes in body composition [128, 129]. Metabolic adaptation to calorie restriction appears to be mediated by reductions in lean mass, sympathetic activity, levels of leptin, insulin, and thyroid hormones, and by greater efficiency of mitochondrial energy metabolism [130].

More recently, some evidence has emerged regarding the possible role of chronic systemic inflammation in promoting weight regain, starting from the observation that the inflammatory imprinting, both cellular and humoral, characterizing obesity, remains unchanged even after weight loss [131–134], determining the so-called “inflammatory memory” of obesity [135], to which the immunometabolic and epigenetic reprogramming of bone marrow myeloid precursors may also contribute [133, 136, 137]. However, the inflammatory memory of obesity, while potentially representing a facilitating factor, does not appear sufficient to explain weight regain in the absence of chronic positive energy balance. Therefore, it is believed that weight regain is based on the same neuro-behavioral pathophysiological mechanisms that characterize obesity [138].

Several clinical trials have demonstrated the phenomenon of “weight regain” upon discontinuation of pharmacological treatment. In STEP 1, after discontinuation of semaglutide, participants regained on average two-thirds of the lost weight, and weight regain continued until the end of follow-up (120 weeks). Nevertheless, some treatment effects persisted, and body weight remained 5.6% lower than baseline. Subgroup analyses showed that patients who had greater weight loss were the same ones who experienced higher weight regain after drug discontinuation, but they were also the ones who remained at a lower body weight at the end of follow-up [139]. Weight regain had previously been observed in other trials following discontinuation of drugs such as orlistat and lorcaserin [117, 140]. Taken together, these data on weight regain related to multiple anti-obesity drugs confirm the chronic nature of the disease and emphasize the importance of long-term pharmacotherapy maintenance. Some pathogenetic mechanisms of body weight regain may play a more specific role after bariatric surgery. Among these are the progressive “normalization” of gastrointestinal hormone levels that are favorably modified by bariatric surgery, the loss of effectiveness of the intervention due to anatomical causes, dumping syndrome that could lead to the need for frequent meals with excessive calorie intake, the development of psychiatric disorders, non-adherence to dietary guidelines, and physical inactivity [141–143]. Approximately 20–25% of patients undergoing bariatric surgery experience weight regain after reaching the nadir of weight loss, which typically occurs between 18 and 24 months after surgery. Furthermore, insufficient weight loss (less than 50%) is the most common cause of surgical revision years after the intervention. The main predictors of weight regain or insufficient weight loss are older age and higher BMI before surgery, male gender, eating behavior disorders, and obesity-related comorbidities [144]. Identifying some predisposing factors to weight regain or insufficient weight loss could improve long-term outcomes. It is also important to emphasize that weight regain is associated with deterioration in quality of life and the recurrence or worsening of obesity-associated comorbidities, such as hypertension and T2D, which require careful monitoring and appropriate management. Long-term management of weight regain requires a multidisciplinary approach based on cognitive-behavioral therapy with frequent lifestyle counseling, pharmacological approaches approved for obesity, and, in some cases, surgical revisions such as conversions from restrictive procedures to malabsorptive procedures.

## Use of medications in chronic or “cycling” regimens to achieve and maintain weight loss in long-term treatment

Body weight reduction is difficult to sustain in the long term, and therefore, the high risk of regaining lost body weight necessitates combined strategies among the various therapies explored so far, as suggested in current guidelines [145, 146]. When initiating pharmacological therapy for obesity, it is important to assess its efficacy and safety at least monthly for the first 3 months and subsequently at least quarterly. Models derived from published clinical studies consistently show that people who respond early to therapy have improved long-term outcomes in terms of body weight reduction [147, 148], with a much more marked response rate with next-generation obesity pharmacotherapies such as GLP1-RAs and dual agonists [76, 77]. Patients who achieve early weight loss > 5% after 3 months of starting pharmacological therapy should continue long-term treatment. When early weight loss results are modest (< 5% weight loss after 3 months of therapy initiation), the benefits of ongoing treatment should be assessed in the context of glycemic response, availability of other potential therapeutic options, tolerance, and overall treatment burden. To date, all randomized studies evaluating the trajectory of body weight upon discontinuation of drugs used to treat obesity, starting from the STORM study that used sibutramine for 2 years [149], have consistently shown significant weight regain upon therapy cessation [150]. Among these, the results of the SCALE study also demonstrated that the combination of Liraglutide 3.0 mg with lifestyle intervention resulted in maintaining the weight loss achieved with calorie restriction and promoted further body weight reduction over 56 weeks of treatment [151]. The STEP4 study further confirmed the need to continue weekly therapy with semaglutide 2.4 mg in promoting long-term weight loss maintenance in people living with overweight/obesity, showing continued and persistent weight loss compared to participants transitioning to placebo and regaining body weight [150]. Finally, the results of the recent SURMOUNT-4 study underline the need to continue tirzepatide to prevent weight regain and ensure maintenance of weight reduction and associated cardiometabolic benefits [152].

Continuation of therapy or the addition of other anti-obesity drugs such as naltrexone/bupropion and orlistat may be used to maintain achieved weight reduction. New studies are needed to build therapeutic algorithms demonstrating the effectiveness and safety of weight control strategy, using where necessary also the alternation between different pharmacological principles as for other chronic diseases. In summary, various classes of drugs, including those that have demonstrated a potent effect on weight loss, have shown that body weight is substantially regained after therapy discontinuation. Therefore, current scientific evidence consistently indicates that, after achieving clinically significant weight reduction, people living with overweight or obesity should continue pharmacological treatment at the maximum tolerated dose to avoid the expected and demonstrated weight regain. At present, scientific literature does not provide any indication for cyclic use of anti-obesity drugs in people who have achieved significant weight loss. Pending further scientific evidence, if the quantitative measure defining significant weight loss is at least 5% compared to baseline, this value could be considered clinically suggestive of weight regain and represent an indication for “cyclic” resumption of pharmacological therapy at the maximum tolerated dose.

## Conclusion

During the UKPDS (UK Prospective Diabetes Study) era, we learned that the natural history of T2D involved a progressive and irreversible deterioration of β-cell function leading to its complete exhaustion and the subsequent need for intensive insulin treatment. Today, under the overwhelming weight of numerous studies on T2D remission after bariatric surgery or VLCD and on the role of adiposopathy (even with mild caloric surplus) with consequent lipotoxicity resembling what occurs in lipodystrophies, the dogma of irreversibility is shattered. We are also at the dawn of a new era characterized by anti-hyperglycemic medications with considerable efficacy in reducing body weight, which could, therefore, play a pathogenetic role in remission of T2D not due to the classic incretin effect but rather due to improvement of adipose tissue functions. All these concepts are elaborately discussed in the paragraphs of this position statement, which represents, in our opinion, a necessary innovative document that shifts the paradigm of “simple” insulin resistance/deficiency interaction. We therefore hope that, having made the “turnaround,” we embark on a new navigation that may lead people living with T2D to the safe harbor of disease remission.
